# Supplementary material for: Genetic imputation of kidney transcriptome, proteome and multi-omics illuminates new blood pressure and hypertension targets
Source: Nat Commun. 2024 Mar 19;15:2359. doi: 10.1038/s41467-024-46132-y (PMC10950894; doi:10.1038/s41467-024-46132-y)
Supplement: Supplementary file 1 — Supplementary information [file 41467_2024_46132_MOESM1_ESM.pdf]

Genetic imputation of kidney transcriptome, proteome and multi-omics illuminates new blood pressure and hypertension targets

Supplementary information

Xiaoguang Xu[1], Chachrit Khunsriraksakul[2], James M. Eales[1], Sebastien Rubin[1], David Scannali[1], Sushant Saluja[1], David Talavera[1], Havell Markus[2], Lida Wang[2], Maciej Drzal[1], Akhlaq Maan[1], Abigail Lay[1], Priscilla R. Prestes[3], Jeniece Regan[2], Avantika R. Diwadkar[2], Matthew Denniff[4], Grzegorz Rempega[5], Jakub Ryszawy[5], Robert Król[6], John P. Dormer[7], Monika Szulinska[8], Marta Walczak[9], Andrzej Antczak[10], Pamela R. Matías-García[11,12,13], Melanie Waldenberger[11,12,13], Adrian S. Woolf[14,15], Bernard Keavney[1,16], Ewa Zukowska-Szczechowska[17], Wojciech Wystrychowski[6], Joanna Zywiec[18], Pawel Bogdanski[8], A.H. Jan Danser[19], Nilesh J. Samani[4], Tomasz J. Guzik[20,21,22], Andrew P. Morris[23], Dajiang J. Liu[2], Fadi J. Charchar[3,4,24], Human Kidney Tissue Resource Study Group and Maciej Tomaszewski[1,16]

[1] Division of Cardiovascular Sciences, Faculty of Medicine, Biology and Health, University of Manchester, Manchester, UK
[2] Department of Public Health Sciences, Penn State College of Medicine, Hershey, PA, USA
[3] Health Innovation and Transformation Centre, Federation University Australia, Ballarat, Australia
[4] Department of Cardiovascular Sciences, University of Leicester, Leicester, UK
[5] Department of Urology, Medical University of Silesia, Katowice, Poland
[6] Department of General, Vascular and Transplant Surgery, Faculty of Medical Sciences in Katowice, Medical University of Silesia, Katowice, Poland
[7] Department of Cellular Pathology, University Hospitals of Leicester, Leicester, UK
[8] Department of Obesity, Metabolic Disorders Treatment and Clinical Dietetics, Karol Marcinkowski University of Medical Sciences, Poznan, Poland
[9] Department of Internal Diseases, Metabolic Disorders and Arterial Hypertension, Poznan University of Medical Sciences, Poznan, Poland
[10] Department of Urology and Uro-oncology, Karol Marcinkowski University of Medical Sciences, Poznan, Poland
[11] Institute of Epidemiology, Helmholtz Center Munich, Neuherberg, Germany
[12] Research Unit Molecular Epidemiology, Helmholtz Center Munich, Neuherberg, Germany
[13] German Research Center for Cardiovascular Disease (DZHK), partner site Munich Heart Alliance, Munich, Germany
[14] Division of Cell Matrix Biology and Regenerative Medicine, Faculty of Biology, Medicine and Health, University of Manchester, Manchester, UK
[15] Royal Manchester Children's Hospital and Manchester Academic Health Science Centre, Manchester University NHS Foundation Trust, Manchester, UK
[16] Manchester Academic Health Science Centre, Manchester University NHS Foundation Trust Manchester, Manchester Royal Infirmary, Manchester, UK
[17] Department of Health Care, Silesian Medical College, Katowice, Poland
[18] Department of Internal Medicine, Diabetology and Nephrology, Zabrze, Medical University of Silesia, Katowice, Poland
[19] Department of Internal Medicine, Division of Pharmacology and Vascular Medicine, Erasmus Medical Centre, Rotterdam, The Netherlands
[20] Department of Internal Medicine, Jagiellonian University Medical College, Kraków, Poland
[21] Centre for Cardiovascular Sciences, Queen's Medical Research Institute, University of Edinburgh, Edinburgh, UK
[22] Center for Medical Genomics OMICRON, Jagiellonian University Medical College, Kraków, Poland
[23] Centre for Genetics and Genomics Versus Arthritis, Centre for Musculoskeletal Research, Division of Musculoskeletal & Dermatological Sciences, Faculty of Medicine, Biology and Health, University of Manchester, Manchester, UK

[24] Department of Physiology, University of Melbourne, Melbourne, Australia

Correspondence:
Professor Maciej Tomaszewski, Division of Cardiovascular Sciences, Faculty of Medicine, Biology and Health, University of Manchester, Manchester, UK
E-mail: maciej.tomaszewski@manchester.ac.uk

Table of Contents

Figure S1. Names for tissue icons shown in Figure 2A 4

Figure S2. Representation of 49 human tissues and cells ranked by the overall systolic (in red) and diastolic (in blue) blood pressure scores of relevance to blood pressure, separately 5

Figure S3. Analytical pipeline and results for transcriptome wide association study of blood pressure 6

Figure S4. Analytical pipeline and the results of Mendelian randomisation and FOCUS analyses 7

Figure S5. Normalised expression of *AGMAT* in the Human Protein Atlas dataset. 8

Figure S6. Effects of genetically regulated expression of *AGMAT* on blood urea nitrogen (BUN) in UK Biobank and CKDGen Consortium 9

Figure S7. Analytical pipeline and the results of kidney microRNA transcriptome-wide association study of blood pressure 10

Figure S8. Analytical pipeline and the results of kidney proteome-wide association study of blood pressure 11

Figure S9. Distribution of RNA-sequencing quality control metrics for 33 urinary cell (yellow) and 40 saliva (blue) samples 12

Human Kidney Tissue Resource Study Group 13

Adipose tissue	Adipose Subcutaneous	(44)
	Adipose Visceral Omentum	(48)
Blood	Whole Blood	(40)
Brain	Brain Amygdala	(42)
	Brain Anterior cingulate cortex BA24	(17)
	Brain Caudate basal ganglia	(18)
	Brain Cerebellar Hemisphere	(9)
	Brain Cerebellum	(10)
	Brain Cortex	(11)
	Brain Frontal Cortex BA9	(4)
	Brain Hippocampus	(20)
	Brain Hypothalamus	(39)
	Brain Nucleus accumbens basal ganglia	(24)
	Brain Putamen basal ganglia	(34)
	Brain Spinal cord cervical c-1	(16)
	Brain Substantia nigra	(35)
Cardiovascular system	Artery Aorta	(5)
	Artery Coronary	(27)
	Artery tibial	(13)
	Heart Atrial Appendage	(46)
	Heart Left Ventricle	(14)
Endocrine system	Adrenal Gland	(6)
	Pituitary	(32)
	Thyroid	(8)
Female reproductive system	Breast Mammary Tissue	(41)
	Ovary	(29)
	Uterus	(33)
	Vagina	(31)
Gastrointestinal system	Colon Sigmoid	(38)
	Colon Transverse	(26)
	Esophagus Gastroesophageal Junction	(43)
	Esophagus Mucosa	(23)
	Esophagus Muscularis	(49)
	Minor Salivary Gland	(12)
	Liver	(15)
	Pancreas	(22)
	Small Intestine Terminal Ileum	(19)
	Stomach	(47)
Immune system	Cells EBV-transformed lymphocytes	(3)
	Spleen	(7)
Male reproductive system	Prostate	(28)
	Testis	(21)
Others	Cells Cultured fibroblasts	(1)
	Muscle Skeletal	(30)
	Nerve Tibial	(37)
Renal system	Kidney Cortex	(2)
Respiratory system	Lung	(45)
Skin	Skin Not Sun Exposed Suprapubic	(36)
	Skin Sun Exposed Lower leg	(25)

Figure S1. Names for tissue icons shown in Figure 2A. Tissues are numbered from 1 to 49 based on their position in the ranking (1 being the top-ranking tissue). 1 – Cells Cultured fibroblasts, 2 – Kidney Cortex, 3 – Cells EBV-transformed lymphocytes, 4 – Brain Frontal Cortex BA9, 5 – Artery Aorta, 6 – Adrenal Gland, 7 – Spleen, 8 – Thyroid, 9 – Brain Cerebellar Hemisphere, 10 – Brain Cerebellum, 11 – Brain Cortex, 12 – Minor Salivary Gland, 13 – Artery Tibial, 14 – Heart Left Ventricle, 15 – Liver, 16 – Brain Spinal cord cervical c-1, 17 – Brain Anterior cingulate cortex BA24, 18 – Brain Caudate basal ganglia, 19 – Small Intestine Terminal Ileum, 20 – Brain Hippocampus, 21 – Testis, 22 – Pancreas, 23 – Esophagus Mucosa, 24 – Brain Nucleus accumbens basal ganglia, 25 – Skin Sun Exposed Lower leg, 26 – Colon Transverse, 27 – Artery Coronary, 28 – Prostate, 29 – Ovary, 30 – Muscle Skeletal, 31 – Vagina, 32 – Pituitary, 33 – Uterus, 34 – Brain Putamen basal ganglia, 35 – Brain Substantia nigra, 36 – Skin Not Sun Exposed Suprapubic, 37 – Nerve Tibial, 38 – Colon Sigmoid, 39 – Brain Hypothalamus, 40 – Whole Blood, 41 – Breast Mammary Tissue, 42 – Brain Amygdala, 43 – Esophagus Gastroesophageal Junction, 44 – Adipose Subcutaneous, 45 – Lung, 46 – Heart Atrial Appendage, 47 – Stomach, 48 – Adipose Visceral Omentum, 49 – Esophagus Muscularis

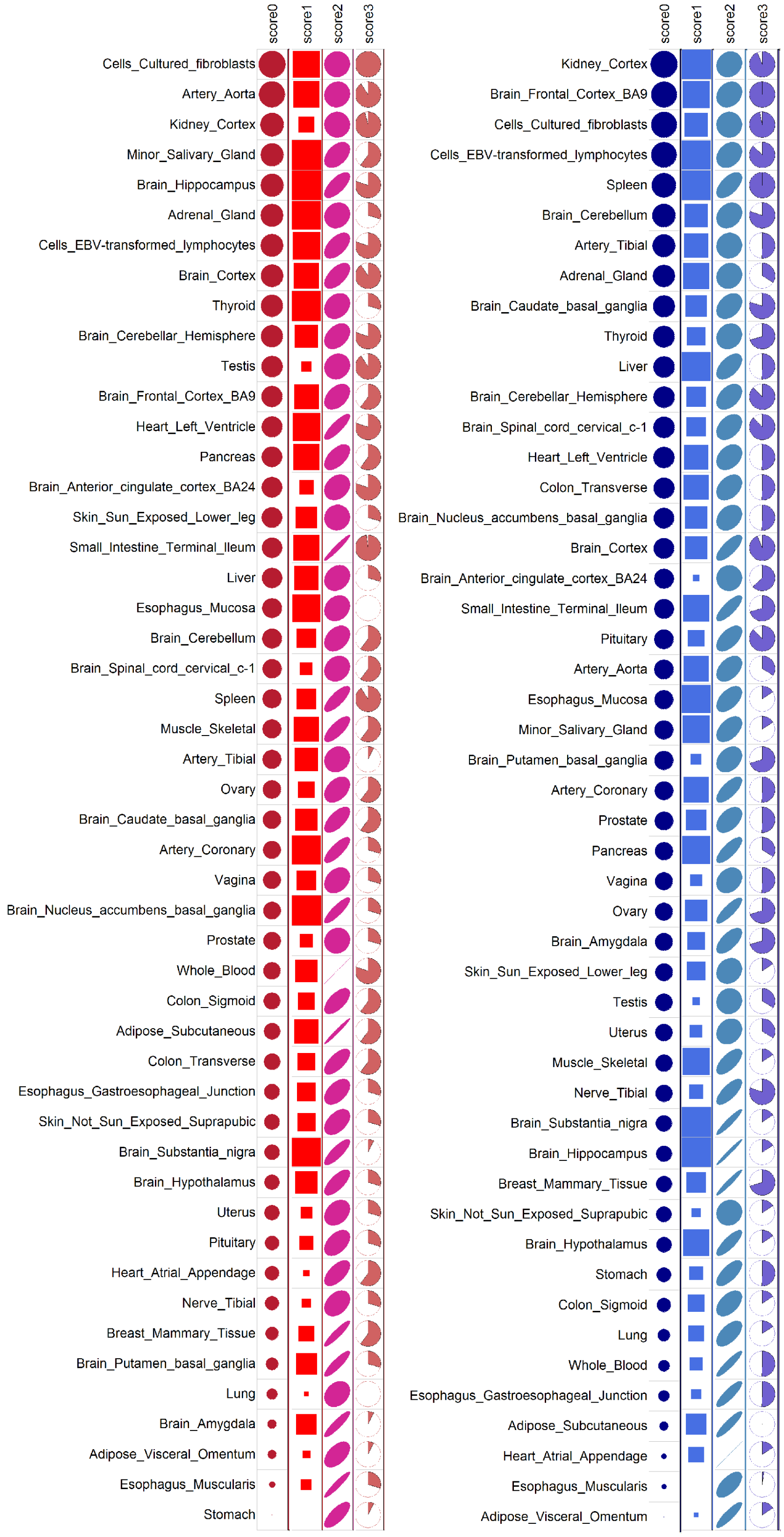

Figure S2. Representation of 49 human tissues and cells ranked by the overall systolic (in red) and diastolic (in blue) blood pressure scores of relevance to blood pressure, separately. Higher scores (i.e. bigger area) represent stronger associations with blood pressure. Score1, Score2 and Score3 represent proportion of independent TWAS signals, average association strength and number of TWAS genes outside GWAS loci respectively. Score0 is sum of Score1, Score2 and Score3.

Blood pressure kidney transcriptome-wide association studies

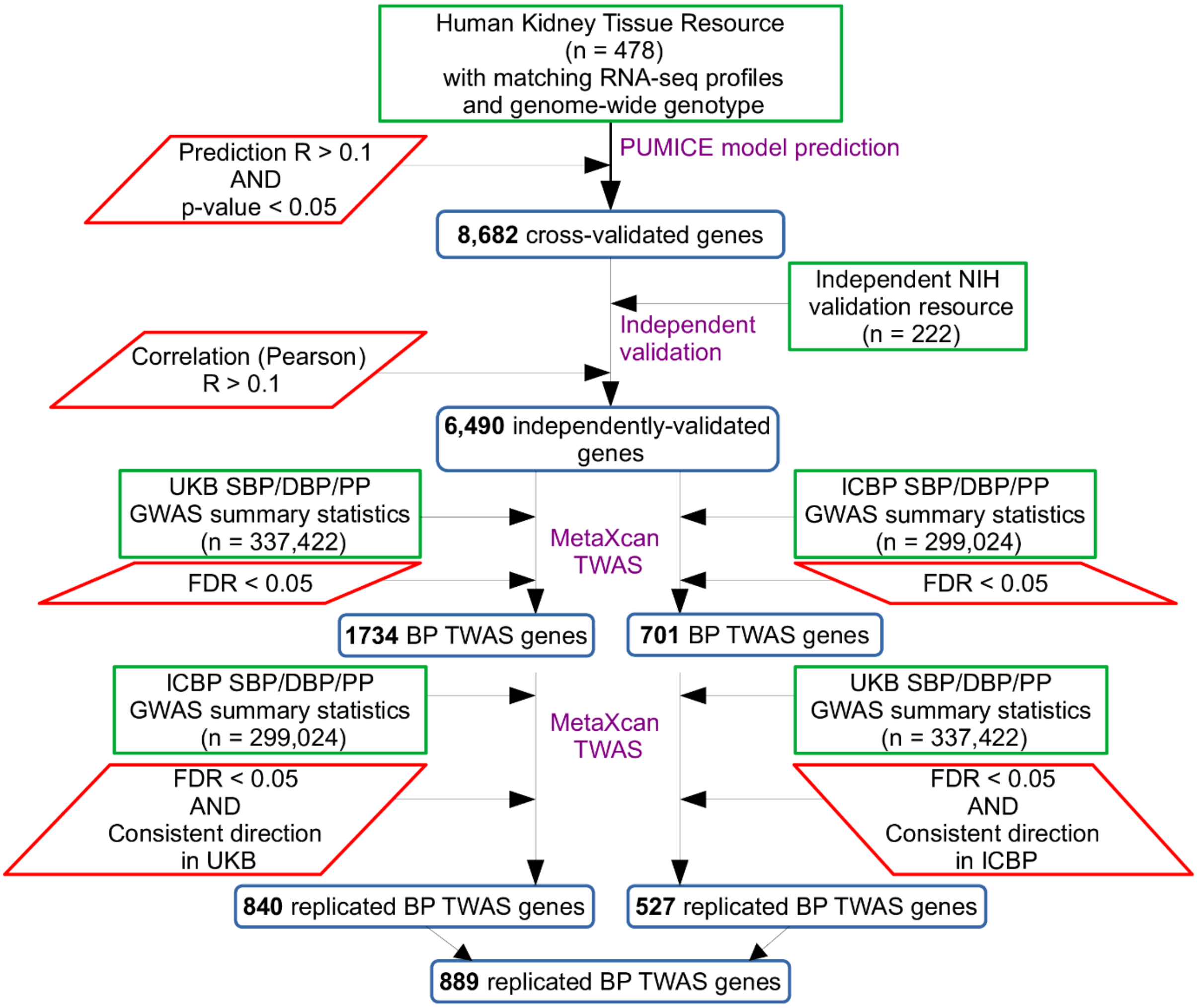

Figure S3. Analytical pipeline and results for transcriptome wide association study of blood pressure. The input data from Human Kidney Tissue Resource (HKTR) and National Institutes of Health (NIH) resources are marked in green boxes, the primary outputs in each analytical step are marked in blue boxes, methods used in each analytical step are coloured in purple and the criteria used in each analytical step are marked in red boxes. n – sample size, R – correlation coefficient, P-value – level of statistical significance, FDR – false discovery rate, UKB – UK Biobank, ICBP – International Consortium for Blood Pressure, TWAS – transcriptome wide association study, BP – blood pressure, SBP – systolic blood pressure, DBP – diastolic blood pressure, PP – pulse pressure.

TWAS, MR and FOCUS prioritised genes mapped to BP GWAS loci

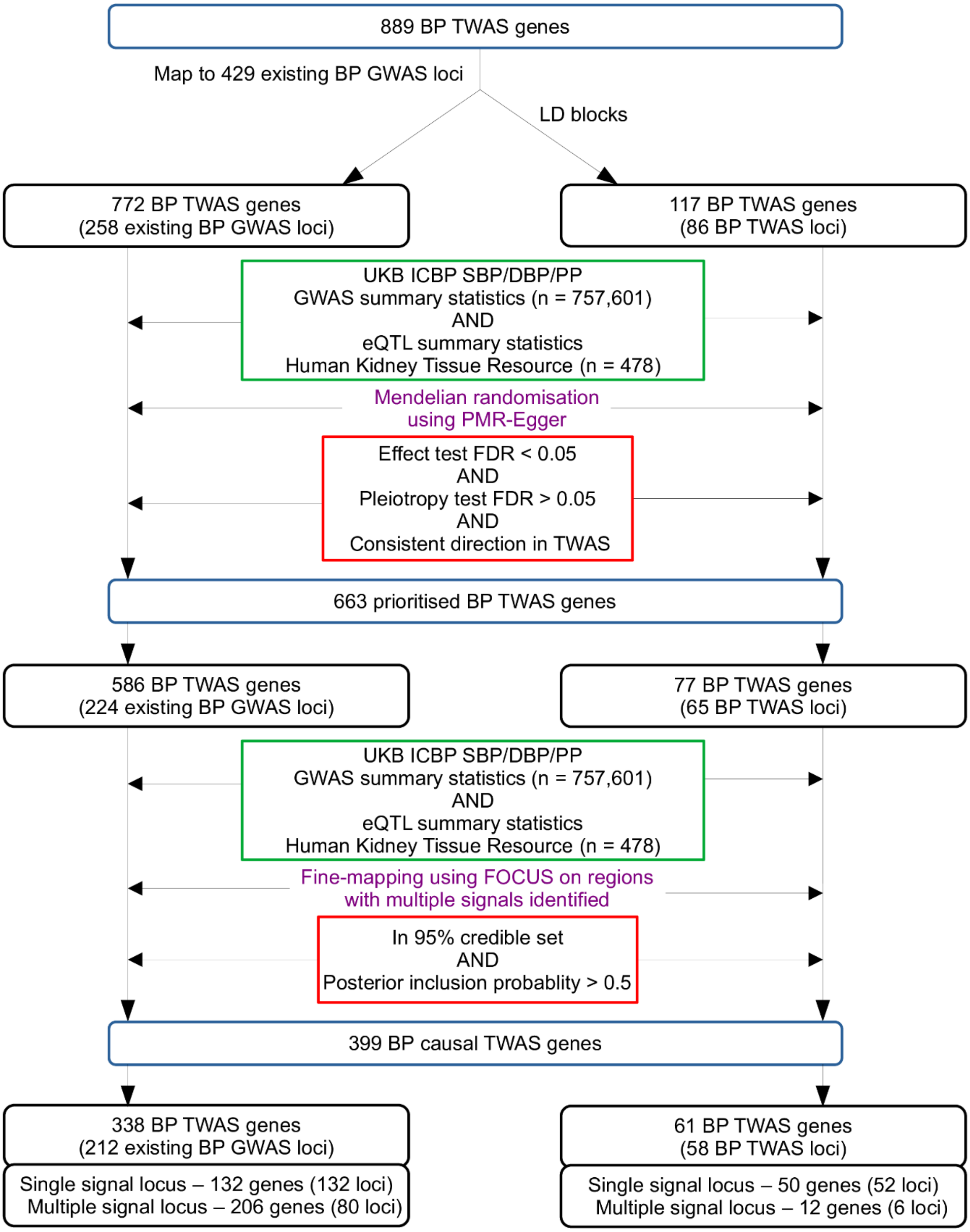

Figure S4. Analytical pipeline and the results of Mendelian randomisation and FOCUS analyses. The input data are marked in green boxes, the primary outputs in each analytical step are marked in blue boxes, methods used in each analytical step are coloured in purple and the criteria used in each analytical step are marked in red boxes. Genes mapped to BP GWAS loci or regions outside BP GWAS loci (i.e. pre-defined LD blocks) are marked in black boxed. n – sample size, FDR – false discovery rate, UKB – UK Biobank, ICBP – International Consortium for Blood Pressure, TWAS – transcriptome wide association study, BP – blood pressure, SBP – systolic blood pressure, DBP – diastolic blood pressure, PP – pulse pressure.

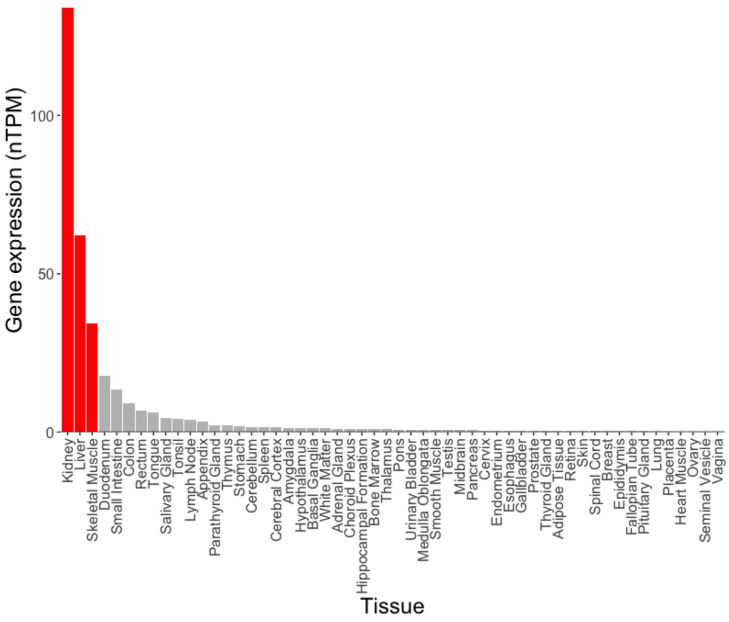

Figure S5. Normalised expression of *AGMAT* in the Human Protein Atlas dataset. Tissues identified as group enriched for this gene are highlighted in red. nTPM – consensus normalised expression in units of transcripts per million.

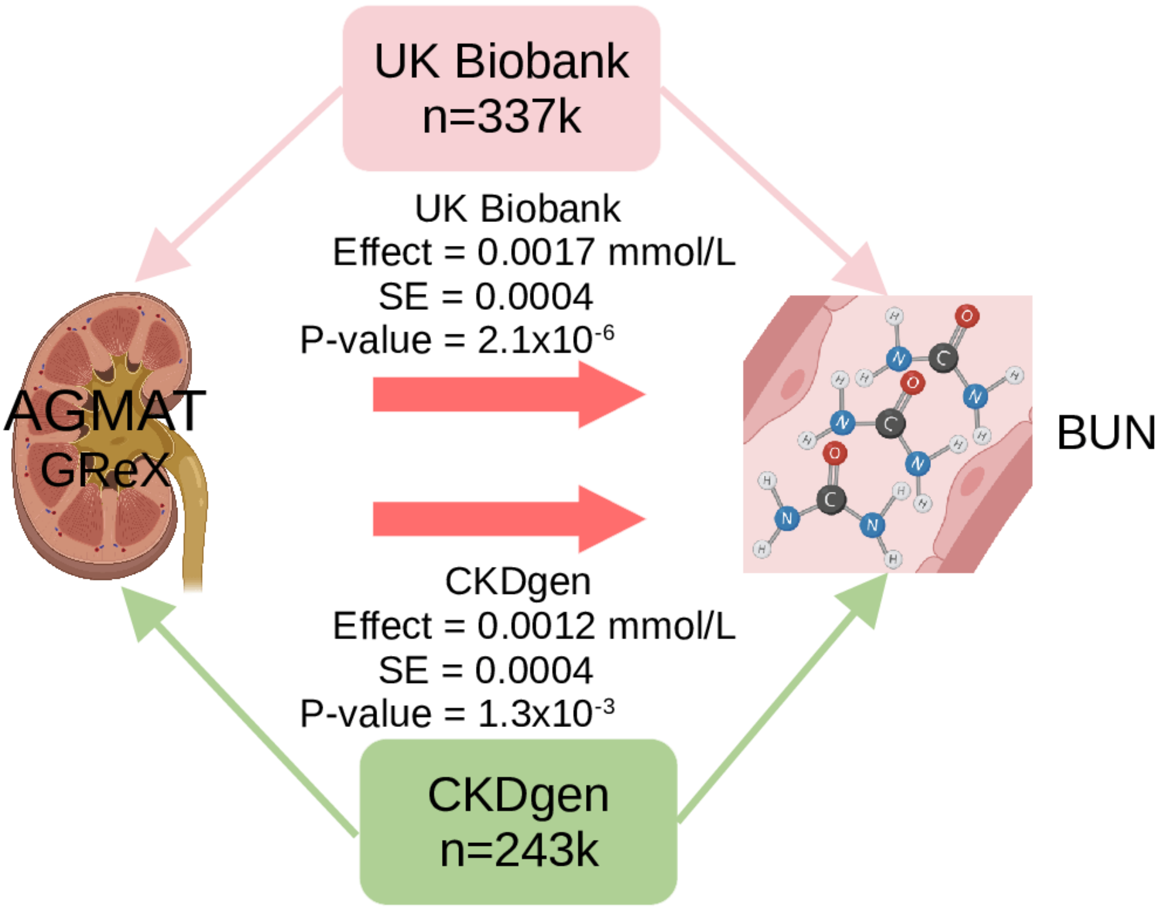

Figure S6. Effects of genetically regulated expression of *AGMAT* on blood urea nitrogen (BUN) in UK Biobank and CKDGen Consortium. GReX – genetically regulated expression, n – sample size, Effect – estimated effect size from AGMAT GReX to BUN (mmol/L change in BUN per one unit higher GReX of *AGMAT*), SE – standard error of the estimated effect size, P-value – level of statistical significance (two-sided z-score test). Partially created with BioRender.com.

Blood pressure kidney miRNA transcriptome-wide association studies

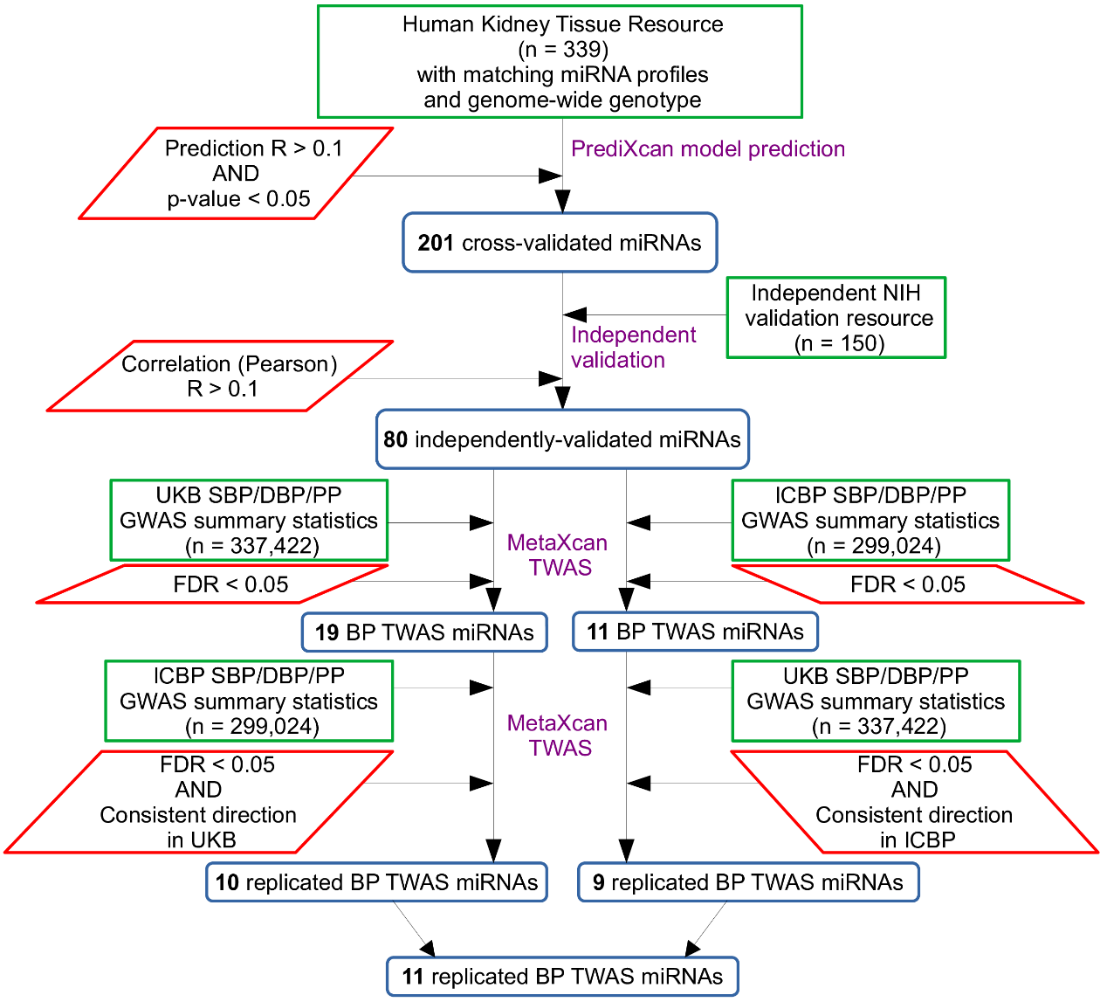

Figure S7. Analytical pipeline and the results of kidney microRNA transcriptome-wide association study of blood pressure. The input data from Human Kidney Tissue Resource (HKTR) and other resources are marked in green boxes, the primary outputs in each analytical step are marked in blue boxes, methods used in each analytical step are coloured in purple and the criteria used in each analytical step are marked in red boxes. miRNA – microRNA, n – sample size, R – correlation coefficient, p-value – level of statistical significance, FDR – false discovery rate, NIH – National Institutes of Health, UKB – UK Biobank, ICBP – International Consortium for Blood Pressure, TWAS – transcriptome wide association study, BP – blood pressure, SBP – systolic blood pressure, DBP – diastolic blood pressure, PP – pulse pressure.

Blood pressure kidney proteome-wide association studies

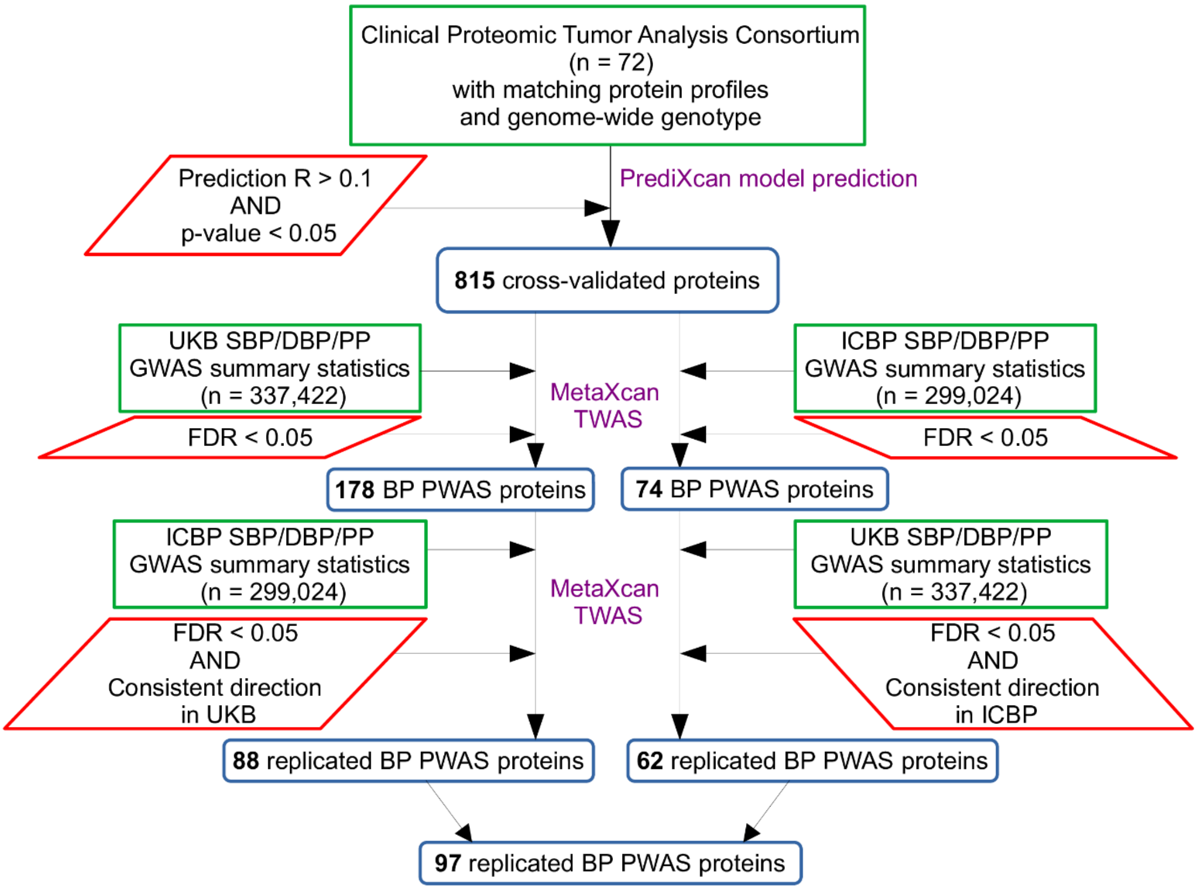

Figure S8. Analytical pipeline and the results of kidney proteome-wide association study of blood pressure. The input data from Clinical Proteomic Tumor Analysis Consortium (CPTAC) and other resources are marked in green boxes, the primary outputs in each analytical step are marked in blue boxes, methods used in each analytical step are coloured in purple and the criteria used in each analytical step are marked in red boxes. n – sample size, R – correlation coefficient, p-value – level of statistical significance, FDR – false discovery rate, NIH – National Institutes of Health, UKB – UK Biobank, ICBP – International Consortium for Blood Pressure, PWAS – proteome wide association study, BP – blood pressure, SBP – systolic blood pressure, DBP – diastolic blood pressure, PP – pulse pressure.

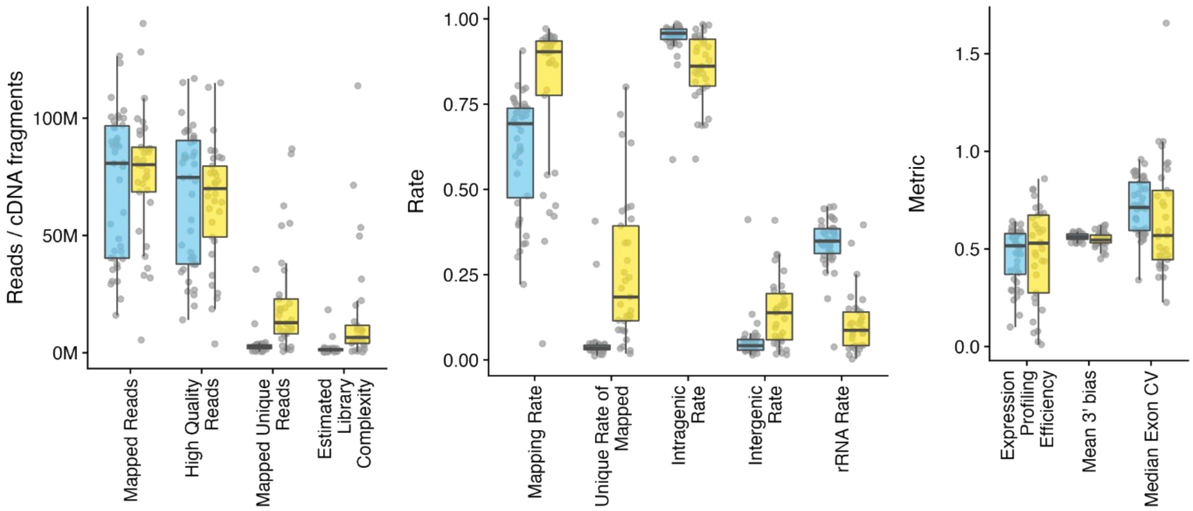

Figure S9. Distribution of RNA-sequencing quality control metrics for 33 urinary cell (yellow) and 40 saliva (blue) samples. All observed metric values are calculated by RNASeQC and are shown as grey points. Whiskers denote the extent of 1.5x interquartile range. Upper, middle, and lower boxplot lines denote 75th, 50th and 25th percentiles, respectively. Boxplots are coloured by sample type; blue – saliva, yellow – urine.

Human Kidney Tissue Resource Study Group:

Maciej Glyda[1], Grzegorz Rempega[2], Jakub Ryszawy[2], Robert Król[3], Monika Szulinska[4], Marta Walczak[5], Andrzej Antczak[6], Bernard Keavney[7,8], Ewa Zukowska-Szczechowska[9], Wojciech Wystrychowski[3], Joanna Zywiec[10], Pawel Bogdanski[4], Fadi J. Charchar[11,12,13], Maciej Tomaszewski[7,8]

[1] Department of Transplantology and General Surgery Poznan, Collegium Medicum, Nicolaus Copernicus University, Bydgoszcz, Poland
[2] Department of Urology, Medical University of Silesia, Katowice, Poland
[3] Department of General, Vascular and Transplant Surgery, Faculty of Medical Sciences in Katowice, Medical University of Silesia, Katowice, Poland
[4] Department of Obesity, Metabolic Disorders Treatment and Clinical Dietetics, Karol Marcinkowski University of Medical Sciences, Poznan, Poland
[5] Department of Internal Diseases, Metabolic Disorders and Arterial Hypertension, Poznan University of Medical Sciences, Poznan, Poland
[6] Department of Urology and Uro-oncology, Karol Marcinkowski University of Medical Sciences, Poznan, Poland
[7] Division of Cardiovascular Sciences, Faculty of Medicine, Biology and Health, University of Manchester, Manchester, UK
[8] Manchester Academic Health Science Centre, Manchester University NHS Foundation Trust Manchester, Manchester Royal Infirmary, Manchester, UK
[9] Department of Health Care, Silesian Medical College, Katowice, Poland
[10] Department of Internal Medicine, Diabetology and Nephrology, Zabrze, Medical University of Silesia, Katowice, Poland
[11] Health Innovation and Transformation Centre, Federation University Australia, Ballarat, Australia
[12] Department of Cardiovascular Sciences, University of Leicester, Leicester, UK
[13] Department of Physiology, University of Melbourne, Melbourne, Australia

Nominated representative:
Professor Maciej Tomaszewski, Division of Cardiovascular Sciences, Faculty of Medicine, Biology and Health, University of Manchester, Manchester, UK
E-mail: maciej.tomaszewski@manchester.ac.uk
