# Supplementary material for: Genetic imputation of kidney transcriptome, proteome and multi-omics illuminates new blood pressure and hypertension targets
Source: Nat Commun. 2024 Mar 19;15:2359. doi: 10.1038/s41467-024-46132-y (PMC10950894; doi:10.1038/s41467-024-46132-y)
Supplement: Supplementary file 3 — Description of Additional Supplementary Files [file 41467_2024_46132_MOESM3_ESM.pdf]

Description of Additional Supplementary Files

File Name: Supplementary Data 1
Description: Tissue prioritisation analysis - systolic blood pressure. Tissues were ordered by the relevance to SBP (from the most relevant to the least relevant). Total score – sum of three scores, twas_ind_signal_proportion – proportion of independent TWAS signals, avg_association_strength – average effect of all the independent TWAS genes on SBP, num_of_genes_outside_gwas_loci – number of genes identified outside existing BP GWAS loci, Sample size – sample size of TWAS analysis from each tissue.

File Name: Supplementary Data 2
Description: Tissue prioritisation analysis - diastolic blood pressure. Tissues were ordered by the relevance to DBP (from the most relevant to the least relevant). Total score – sum of three scores, twas_ind_signal_proportion – proportion of independent TWAS signals, avg_association_strength – average effect of all the independent TWAS genes on DBP, num_of_genes_outside_gwas_loci – number of genes identified outside existing BP GWAS loci, Sample size – sample size of TWAS analysis from each tissue.

File Name: Supplementary Data 3
Description: Kidney gene expression predictive models generated by PUMICE (Prediction Using Models Informed by Chromatin conformations and Epigenomics) and PrediXcan. Method – gene expression prediction algorithm, HKTR – Human Kidney Tissue Resource, NIH – National Institutes of Health, n – sample size.

File Name: Supplementary Data 4
Description: 6490 independently validated kidney gene expression predictive models generated by PUMICE (Prediction Using Models Informed by Chromatin conformations and Epigenomics). Discovery panel (n=478) - Human Kidney Tissue Resource. Validation panel (n=222) - NIH kidney resources. P-value – nominal P-value calculated from two-sided Pearson correlation, lncrna – long non-coding RNA. R - Pearson correlation coefficient between predicted expression and observed expression.

File Name: Supplementary Data 5
Description: Analysis of association between predicted kidney expression of metabolic transporters/receptors and the relevant blood biochemistry and diseases in up to 337,350 individuals from UK Biobank. n – number of individuals, P-value – nominal P-value calculated from linear regression (two-sided test) for quantitative traits and from logistic regression (two-sided test) for binary traits, nCases – number of cases, nControls – number of controls, FDR – false discovery rate.

File Name: Supplementary Data 6
Description: Predicted expression of 889 kidney genes associated with blood pressure traits. Each BP TWAS locus from 1 to 429 is mapped to each of the 429 re-defined BP GWAS loci. Any BP TWAS locus from 430 is mapped to pre-defined LD blocks not overlapped with any BP GWAS locus. SBP – systolic blood pressure, DBP – diastolic blood pressure, PP – pulse pressure, P-value – nominal P-value calculated from two-sided Z-score test, FDR – false discovery rate, Beta – mmHg changes in the outcome trait per one standard deviation increase of the predicted expression of the gene. SE – standard error of Beta, 95%CIL – 95% confidence interval lower boundary, 95%CIU – 95% confidence interval upper boundary, UKB – study

using UK Biobank, ICBP – study using International Consortium for Blood Pressure, UKB+ICBP – study using combined UKB and ICBP.

File Name: Supplementary Data 7
Description: Kidney genes uncovered by BP kidney TWAS, not amongst BP genes from previous TWAS (Giri, A. et al. Trans-ethnic association study of blood pressure determinants in over 750,000 individuals. Nat. Genet. 51, 51–62 (2019), Wu, P. et al. Integrating gene expression and clinical data to identify drug repurposing candidates for hyperlipidemia and hypertension. Nat. Commun. 13, 46 (2022)) and not amongst kidney function genes from previous TWAS (Schlosser et al. Transcriptome- and proteome-wide association studies nominate determinants of kidney function and damage. Genome Biol. 24, 150 (2023)). TWAS genes identified by previous studies are numbered 1, otherwise, 0. TWAS – transcriptome-wide association study, FDR significant – TWAS genes identified after FDR correction for multiple testing at corrected P-value < 0.05.

File Name: Supplementary Data 8
Description: Computational drug repurposing results for each drug classes stratified by the ability to reverse / induce high blood pressure traits. The example of drugs in each drug class are listed. A/B refers to number of drug-cell pairs with negative (<-75) / positive (>75) tau scores, respectively. Nominal P-value is calculated using two-sided binomial test applied to the "Combined" result. Results are organized by the P-value from lowest to highest. Directions are annotated as "induce" if the drug class has more drug-cell pairs with positive tau scores (i.e., increasing blood pressure) and "reverse" if drug class has more drug-cell pairs with negative tau scores (i.e., decreasing blood pressure). SBP – systolic blood pressure, DBP – diastolic blood pressure, PP – pulse pressure, FDR – false discovery rate.

File Name: Supplementary Data 9
Description: Causal effect of genetically regulated expression of kidney genes on blood pressure traits from PMR-Egger. Each BP TWAS locus from 1 to 429 is mapped to each of the 429 re-defined BP GWAS loci. Any BP TWAS locus from 430 is mapped to pre-defined LD blocks not overlapped with any BP GWAS locus. TWAS – transcriptome-wide association study, SBP – systolic blood pressure, DBP – diastolic blood pressure, PP – pulse pressure, Beta – mmHg changes in the outcome trait per one standard deviation increase of the genetically regulated expression of the gene. SE – standard error of Beta, 95%CIL – 95% confidence interval lower boundary, 95%CIU – 95% confidence interval upper boundary, P-value – nominal P-value is calculated from one-sided chi-squared test, FDR – false discovery rate, nSNP – number of SNPs used in the PMR-Egger analysis. PMR – PMR-Egger.

File Name: Supplementary Data 10
Description: Focus prioritised NMT1 from 10 genes showing causal associations with diastolic blood pressure in the same region on chromosome 17. TWAS – transcriptome-wide association study, In credible set or not – gene selected into a credible set is labelled "Yes", otherwise, "No". lincRNA – long non-coding RNA, DBP – diastolic blood pressure.

File Name: Supplementary Data 11
Description: Overview of 399 genes causally related to blood pressure (BP). Gene symbols, IDs, full names and biotypes are taken from Ensembl v83. The classification process involved capturing top-level Reactome terms, manually mapping terms for genes without annotations, and manually selecting the top-level term when multiple options were available. Data for

tractability analysis were collected from OpenTarget. Tractability includes small molecule, antibody, and PROTAC (Proteolysis-Targeting Chimeras). Tractability druggable family comes from the Druggable Proteome (Human Protein Atlas https://www.proteinatlas.org/humanproteome/tissue/druggable). FINNGEN rare variant – evidence of loss of function and missense variants with minor allele frequency < 5% (in the gene region) associated with traits related to hypertension (P-value < 1E-4) from FinnGen GWAS analysis (r9.finngen.fi). Z-score – Z statistics from kidney BP TWAS in UK Biobank and ICBP (n=750,000).

File Name: Supplementary Data 12
Description: Association between genetically regulated kidney expression of solute carrier family 5 (sodium/inositol cotransporter), member 11 gene (SLC5A11) and glucose/HbA1c in UK Biobank. GReX – genetically regulated expression, Beta – SD changes in urinary sodium per one standard deviation increase of ENPEP GReX, 95%CIL – 95% confidence interval lower boundary, 95%CIU – 95% confidence interval upper boundary, P-value – nominal P-value calculated from linear regression (two-sided test), Field – UK Biobank data field.

File Name: Supplementary Data 13
Description: 80 independently validated kidney microRNA expression predictive models generated by PrediXcan. Discovery panel (n=339) and validation panel (n=150). miRNA – microRNA. P-value – nominal P-value calculated from two-sided Pearson correlation, R - Pearson correlation coefficient between predicted expression and observed expression.

File Name: Supplementary Data 14
Description: Significant associations of kidney microRNAs with blood pressure traits in UK Biobank and International Consortium for Blood Pressure cohorts in a transcriptome-wide association study. Each BP TWAS locus from 1 to 429 is mapped to each of the 429 re-defined BP GWAS loci. miRNA – microRNA, SBP – systolic blood pressure, DBP – diastolic blood pressure, PP – pulse pressure, Beta – mmHg changes in the outcome trait per one standard deviation increase of the predicted expression of the miRNA, P-value – nominal P-value calculated from two-sided Z-score test, FDR – false discovery rate, UKB – study using UK Biobank, ICBP – study using International Consortium for Blood Pressure.

File Name: Supplementary Data 15
Description: Selected characteristics of 11 miRNAs showing significant associations with at least one blood pressure trait by TWAS.

File Name: Supplementary Data 16
Description: Conditional analysis on BP kidney TWAS miRNAs and the neighbouring genes. Matching traits – the same BP trait miRNA and the corresponding gene associated with from microRNA TWAS and TWAS analyses. SBP – systolic blood pressure, DBP – diastolic blood pressure, PP – pulse pressure, UKB – study using UK Biobank, ICBP – study using International Consortium for Blood Pressure, Zscore_miRNA – standardised effect size estimated from microRNA TWAS, Zscore_gene – standardised effect size estimated from TWAS.

File Name: Supplementary Data 17
Description: Protein classification for 7,291 proteins measurable in CPTAC kidney tissue samples. Protein localisation data from Human Protein Atlas (data accessed 02/2022).

File Name: Supplementary Data 18
Description: Enrichment in the Clinical Proteomic Tumor Analysis Consortium (CPTAC) dataset for proteins whose parent genes show enriched or enhanced expression in the human kidney. Hypergeometric test. P-value – nominal P-value is calculated from one-sided hypergeometric test.

File Name: Supplementary Data 19
Description: Correlation between protein abundance and parent gene expression for 7,036 protein/gene pairs derived from the Clinical Proteomic Tumor Analysis Consortium (CPTAC) human kidney tissue proteomics dataset (n=65). r – Pearson correlation coefficient, P-value – nominal P-value calculated from two-sided Pearson correlation, FDR – false discovery rate.

File Name: Supplementary Data 20
Description: Correlation between protein abundance and parent gene expression in human kidney tissue for 152 causal BP TWAS genes, 7 antihypertensive drug targets, 8 monogenic hypertension or hypotension genes and 216 genes with enhanced or enriched expression in kidney tissue from Human Protein Atlas. Protein abundance from the Clinical Proteomic Tumor Analysis Consortium (CPTAC) human kidney tissue proteomics dataset. r – Spearman's correlation coefficient. P-value – calculated from two-sided Spearman's correlation, FDR – p-value corrected for multiple testing using false discovery rate method.

File Name: Supplementary Data 21
Description: 815 cross-validated kidney protein abundance predictive models generated by PrediXcan. Gene – gene which encoded the protein, R – Pearson correlation coefficient between predicted and observed protein abundance, P-value – nominal P-value calculated from two-sided Pearson's correlation.

File Name: Supplementary Data 22
Description: Predicted protein abundance of 97 kidney genes associated with blood pressure traits. SBP – systolic blood pressure, DBP – diastolic blood pressure, PP – pulse pressure, P-value – nominal P-value calculated from two-sided Z-score test, FDR – false discovery rate, UKB – study using UK Biobank, ICBP – study using International Consortium for Blood Pressure.

File Name: Supplementary Data 23
Description: Overview of 97 kidney proteins associated with blood pressure (BP) through kidney proteome-wide association study (PWAS). The biological classification process involved capturing top-level Reactome terms, manually mapping terms for genes without annotations, and manually selecting the top-level term when multiple options were available. Column (Availability of GReX model) shows whether the protein has a GReX prediction model. Column (Prior evidence from kidney omics) contains "twas" – has evidence of association with BP at mRNA level from TWAS analysis, "sQTL" - has evidence of colocalisation with BP at kidney alternative splicing level, "eQTL" - has evidence of colocalisation with BP at kidney mRNA level and "mQTL" - has evidence of colocalisation with BP at kidney DNA methylation level based on the PUMICE-based TWAS analysis or previous kidney QTL studies (Eales et al. Nat Genet. 2021;53:630-637). Z-score – standardised effect size estimated from BP PWAS analysis, UKB – UK Biobank, FDR – false discovery rate.

File Name: Supplementary Data 24
Description: 12 overrepresented KEGG pathways and Human Phenotype Ontology diseases in 97 BP PWAS proteins (hypergeometric test). All results are significant at 5% FDR. Results are ordered by P-value. KEGG – Kyoto encyclopedia of genes and genomes, P-value – nominal P-value calculated from one-sided hypergeometric test, FDR – false discovery rate.

File Name: Supplementary Data 25
Description: Demographic characteristics of individuals who underwent urinary pellet RNA-sequencing. Data are counts and percentages or means and standard deviations. n – total number of samples analysed BMI – body mass index.

File Name: Supplementary Data 26
Description: 68 overrepresented KEGG pathways and Gene Ontology - Biological Process terms in top 100 expressed genes from urinary cells and kidney tissue samples. All results are from DAVID overrepresentation analysis and are significant at 5% FDR. P-value – nominal P-value calculated from one-sided Fisher's exact test, FDR – false discovery rate.

File Name: Supplementary Data 27
Description: GTEx tissue labels used in Figure 7E for 54 GTEx tissues.

File Name: Supplementary Data 28
Description: Nomalised expression of ENPEP across human tissues from The Genotype-Tissue Expression (GTEx) project and The Human Protein Atlas (HPA). nTPM – normalised transcripts per million.

File Name: Supplementary Data 29
Description: Colocalisation for ENPEP kidney expression, kidney protein abundance and diastolic blood pressure. DBP – diastolic blood pressure.

File Name: Supplementary Data 30
Description: Linkage disequilibrium r2 score between rs33966350 and single nucleotide polymorphisms (SNPs) from ENPEP gene expression PUMICE model based on 1000 genome European reference panel. SNP1 – snps from ENPEP gene expression PUMICE model, SNP2 – rs33966350, r2 – linkage disequilibrium score ordered from largest to smallest.

File Name: Supplementary Data 31
Description: Demographic characteristics of individuals from Human Kidney Tissue Resource. Data are counts and percentages or means and standard deviations. HKTR – Human Kidney Tissue Resource, n – number of individuals, BMI – body mass index.

File Name: Supplementary Data 32
Description: Demographic characteristics of individuals from NIH cohorts. Data are counts and percentages or means and standard deviations. TCGA – Human Kidney Tissue Resource, GTEx – The Genotype-Tissue Expression project, CPTAC – Clinical Proteomic Tumor Analysis Consortium, n – number of individuals.

File Name: Supplementary Data 33
Description: Information on genome-wide association studies used as an input into kidney transcriptome-wide association studies on blood pressure. Included were: genome-wide association studies on systolic, diastolic blood pressure and pulse pressure from UK Biobank and International Consortium for Blood Pressure [Evangelou, E. et al. Genetic analysis of over

1 million people identifies 535 new loci associated with blood pressure traits. Nat. Genet. 50, 1412–1425 (2018)]. Genomic inflation factor was estimated using LD score regression intercept (with standard error of the estimate). UKB – study using UK Biobank, ICBP – study using International Consortium for Blood Pressure, MAF – minor allele frequency.

File Name: Supplementary Data 34
Description: 429 existing blood pressure GWAS loci defined using previous blood pressure GWAS SNPs (Evangelou, E. et al. Genetic analysis of over 1 million people identifies 535 new loci associated with blood pressure traits. Nat. Genet. 50, 1412–1425 (2018)). Each BP TWAS locus from 1 to 429 is mapped to each of the 429 re-defined existing blood pressure GWAS loci.

File Name: Supplementary Data 35
Description: Demographic characteristics of individuals from Human Kidney Tissue Resource and NIH resources used for microRNA transcriptome-wide association study. Data are counts and percentages or means and standard deviations. HKTR – Human Kidney Tissue Resource, TCGA – The Cancer Genome Atlas, CPTAC – The Cancer Genome Atlas, NIH – National Institutes of Health, n – number of individuals, BMI – body mass index.

File Name: Supplementary Data 36
Description: Categorisation of Ensembl database gene biotype values into broad gene biotype categories. Ensembl gene biotypes were collected from Ensembl gene BioMart database v83.
