# Supplementary material for: Genetic imputation of kidney transcriptome, proteome and multi-omics illuminates new blood pressure and hypertension targets
Source: Nat Commun. 2024 Mar 19;15:2359. doi: 10.1038/s41467-024-46132-y (PMC10950894; doi:10.1038/s41467-024-46132-y)
Supplement: Supplementary file 5 — Reporting Summary [file 41467_2024_46132_MOESM5_ESM.pdf]

nature portfolio

Corresponding author(s): Maciej Tomaszewski

Last updated by author(s): Jan 9, 2024

Reporting Summary

Nature Portfolio wishes to improve the reproducibility of the work that we publish. This form provides structure for consistency and transparency in reporting. For further information on Nature Portfolio policies, see our Editorial Policies and the Editorial Policy Checklist.

Statistics

For all statistical analyses, confirm that the following items are present in the figure legend, table legend, main text, or Methods section.

n/a	Confirmed	
☐	☒	The exact sample size (*n*) for each experimental group/condition, given as a discrete number and unit of measurement
☒	☐	A statement on whether measurements were taken from distinct samples or whether the same sample was measured repeatedly
☐	☒	The statistical test(s) used AND whether they are one- or two-sided<br>*Only common tests should be described solely by name; describe more complex techniques in the Methods section.*
☐	☒	A description of all covariates tested
☐	☒	A description of any assumptions or corrections, such as tests of normality and adjustment for multiple comparisons
☐	☒	A full description of the statistical parameters including central tendency (e.g. means) or other basic estimates (e.g. regression coefficient) AND variation (e.g. standard deviation) or associated estimates of uncertainty (e.g. confidence intervals)
☐	☒	For null hypothesis testing, the test statistic (e.g. *F*, *t*, *r*) with confidence intervals, effect sizes, degrees of freedom and *P* value noted<br>*Give P values as exact values whenever suitable.*
☒	☐	For Bayesian analysis, information on the choice of priors and Markov chain Monte Carlo settings
☒	☐	For hierarchical and complex designs, identification of the appropriate level for tests and full reporting of outcomes
☐	☒	Estimates of effect sizes (e.g. Cohen's *d*, Pearson's *r*), indicating how they were calculated

Our web collection on statistics for biologists contains articles on many of the points above.

Software and code

Policy information about availability of computer code

Data collection	Custom code was not used to collect data for this work.
Data analysis	R versions 4.2.2., plink version 1.9., FastQC version 0.11.7. RNA-SeQC version 1.1.8., Kallisto version 0.44.0., PEER version 1.3., King version 1.9c., SNPWeights version 2.1., EIGENSTRAT version 6.0.1., FastQTL version 2.184., TwoSampleMR version 0.5.6., MendelianRandomization version 0.7.1., PMR version 1.0., FOCUS version 0.6.10., HyPrColoc version 1.0.0., Seurat version 4.0.3., MuSiC version 1.0.0., Michigan imputation server (accessed June 2019) including minimac4 version 1.7.3. and Eagle version 2.4., PrediXcan, S-PrediXcan, PUMICE version 1.0.0., EMBOSS, MODELLER version 10.4.

For manuscripts utilizing custom algorithms or software that are central to the research but not yet described in published literature, software must be made available to editors and reviewers. We strongly encourage code deposition in a community repository (e.g. GitHub). See the Nature Portfolio guidelines for submitting code & software for further information.

Data

Policy information about availability of data

All manuscripts must include a data availability statement. This statement should provide the following information, where applicable:

- Accession codes, unique identifiers, or web links for publicly available datasets
- A description of any restrictions on data availability
- For clinical datasets or third party data, please ensure that the statement adheres to our policy

The PUMICE-derived TWAS, microRNA-TWAS and PWAS summary statistics generated in this study are available in the Supplementary Data 6, 14 and 22, respectively. Bulk RNA-seq data TCGA, CPTAC and GTEx can be accessed from Genomic Data Commons (GDC) Data Portal (https://portal.gdc.cancer.gov/) and GTEx portal (https://gtexportal.org/home/). The normalised kidney gene expression, microRNA expression data and urinary transcriptomic data from HKTR are archived at https://doi.org/10.48420/24871785. Full summary statistics of blood pressure GWAS using 337,422 unrelated white European individuals from UK Biobank are available at https://doi.org/10.48420/24851436. The sample-size-balanced GTEx v8 TWAS models are available at https://doi.org/10.48420/24871794. The kidney PUMICE model is available at https://github.com/ckhunsr1/PUMICE/tree/master/model_HKTR. Source data are provided with this paper.

Research involving human participants, their data, or biological material

Policy information about studies with human participants or human data. See also policy information about sex, gender (identity/presentation), and sexual orientation and race, ethnicity and racism.

Reporting on sex and gender	Self-reported sex information was collected in the HKTR study, and then confirmed by genotype. The HKTR study contains 478 samples, of these 300 (62.8%) are male. For the NIH studies (i.e. TCGA, GTEx and CPTAC), sample size and sex information can be found in detail in Supplementary Data 32. 337,422 individuals from UK Biobank were used in our study - 156,260 (63%) are male.
Reporting on race, ethnicity, or other socially relevant groupings	White European samples were used in all the studies.
Population characteristics	Participants included both males and females of white European ethnicity. Full demographic details of the HKTR, TCGA, GTEx and CPTAC studies are provided in Supplementary Data 31-32. Demographic details of the UK Biobank cohort is available elsewhere https://bmjopen.bmj.com/content/6/3/e009161.
Recruitment	The HKTR consists of five studies, i.e. TRANSLATE, TRANSLATE-T, ADMIRE, RESPOND and REPAIR. The TRANSLATE study recruited patients diagnosed with unilateral non-invasive renal cancer, eligible for elective nephrectomy and with no apparent history of primary nephropathy. These samples were selected solely on their eligibility and need for elective nephrectomy. TRANSLATE-T samples were collected by pre-implantation biopsy from deceased donors' kidneys prior to transplantation. ADMIRE samples were collected from individuals undergoing elective surgical removal of the kidney because of kidney cancer. RESPOND recruits patients diagnosed with kidney cancer referred for an elective nephrectomy. The renal tissue samples were taken from healthy (unaffected by cancer) pole of the kidney after surgical removal of the organ and processed through the pipeline developed earlier in TRANSLATE study. REPAIR study kidney specimens were collected from renal grafts prior to organ transplantation, tissue samples were obtained by needle biopsy. Samples from TCGA, GTEx and CPTAC were recruited as part of NIH-approved studies to establish a resource with human tissue for further molecular studies. Recruitment details and dataset overviews can be found in https://doi.org/10.1371/journal.pmed.1001779 for UK Biobank.
Ethics oversight	The studies adhered to the Declaration of Helsinki and were approved/ratified by the Bioethics Committee of the Medical University of Silesia (Katowice, Poland), the Bioethics Committee of Karol Marcinkowski Medical University (Poznan, Poland), the Ethics Committee of the University of Leicester (Leicester, UK), the University of Manchester Research Ethics Committee (Manchester, UK) and the National Research Ethics Service Committee North West (Manchester, UK). Informed, written consents were obtained from all individuals recruited (for the deceased donors, the consent was obtained in line with the local governance; for example, from the family members). UK Biobank has approval from the North West Multi-centre Research Ethics Committee (MREC) to obtain and disseminate data and samples from the participants, and these ethical regulations cover the work in this study. Written informed consent was obtained from all participants.

Note that full information on the approval of the study protocol must also be provided in the manuscript.

Field-specific reporting

Please select the one below that is the best fit for your research. If you are not sure, read the appropriate sections before making your selection.

☒ Life sciences ☐ Behavioural & social sciences ☐ Ecological, evolutionary & environmental sciences

For a reference copy of the document with all sections, see nature.com/documents/nr-reporting-summary-flat.pdf

Life sciences study design

All studies must disclose on these points even when the disclosure is negative.

Sample size	Our study is based on a very large collection of human kidneys characterised at multiple molecular levels (n=772). This is the maximal number of samples available to us - we did not pre-determine sample size and relied on maximal number of samples available to us.
Data exclusions	Genotyping data was excluded based on missingness, signs of sample mixing, mixed ethnicity, excess heterozygosity or cryptic relatedness. RNA-sequencing samples were excluded that showed signs of erroneous sex chromosome gene expression, sample mixing or were D-statistic outliers. Full details of all data exclusions are provided in the Methods section. Our exclusion criteria were pre-determined based on our previous work (Eales. et al, PMID: 33958779).
Replication	Prediction performance of PUMICE-derived GReX models in kidney was validated in an independent cohort from NIH studies (i.e. TCGA+GTEx +CPTAC). In addition, we conducted kidney BP TWAS using a reciprocal replication strategy in UK Biobank and ICBP.
Randomization	Our study is not a clinical trial. Therefore, it's not relevant to our study.
Blinding	Our study is not a clinical trial. Therefore, it's not relevant to our study.

Reporting for specific materials, systems and methods

We require information from authors about some types of materials, experimental systems and methods used in many studies. Here, indicate whether each material, system or method listed is relevant to your study. If you are not sure if a list item applies to your research, read the appropriate section before selecting a response.

Materials & experimental systems

n/a	Involved in the study	
☒	☐	Antibodies
☒	☐	Eukaryotic cell lines
☒	☐	Palaeontology and archaeology
☒	☐	Animals and other organisms
☒	☐	Clinical data
☒	☐	Dual use research of concern
☒	☐	Plants

Methods

n/a	Involved in the study	
☒	☐	ChIP-seq
☒	☐	Flow cytometry
☒	☐	MRI-based neuroimaging
